# The prevalence of chronic pain and its associated factors among Saudi Al-Kharj population; a cross sectional study

**DOI:** 10.1186/s12891-019-2555-7

**Published:** 2019-04-25

**Authors:** Ashraf El-Metwally, Quratulain Shaikh, Abdulrahman Aldiab, Jamaan Al-Zahrani, Sameer Al-Ghamdi, Abdullah A. Alrasheed, Mowafa Househ, Omar B. Da’ar, Shanila Nooruddin, Hira Abdul Razzak, Khaled K. Aldossari

**Affiliations:** 1 College of Public Health & Health Informatics, King Saud Bin Abdulaziz University for Health Sciences, Mail Code 2350, P.O. Box 3660, Riyadh, 11481 Kingdom of Saudi Arabia; 2 Docent of Epidemiology, School of Health Sciences, University of Tampere, Tampere, Finland; 3 Indus Hospital Research Center, Indus Health Network, Karachi, Pakistan; 4 Department of Medicine, Oncology division, College of Medicine, King Saud University, Riyadh, Saudi Arabia; 5 Family & Community Medicine Department, College of Medicine, Prince Sattam Bin Abdulaziz University, Al-Kharj, Saudi Arabia; 6 Department of Family Medicine, College of Medicine, Prince Sattam Bin Abdulaziz University, Al-Kharj, Saudi Arabia; 7 Family and Community Medicine Department, College of Medicine, King Saud University, Riyadh, Saudi Arabia; 8 School of Health Information Science, University of Victoria, Victoria, Canada; 9 St. Mary’s University of Minnesota, Winona, MN, USA; 10 Dow University of Health Sciences, Karachi, Pakistan; 11 Ministry of Health and Prevention, Dubai, United Arab Emirates; 12 Family & Community Medicine Department, College of Medicine, Prince Sattam Bin Abdulaziz University, Al-Kharj, Saudi Arabia

**Keywords:** Chronic pain, Back pain, Al Kharj, Saudi Arabia

## Abstract

**Background:**

Chronic pain (CP) can be a symptom of many underlying health issues. The consequences of CP may vary from slight discomfort to disruption of quality of life and normal functioning. In this study, we aim to investigate the prevalence of CP and its associated factors in Al Kharj, Saudi Arabia.

**Methods:**

This was a cross-sectional study conducted in Al Kharj, Saudi Arabia. We recruited 1031 participants for our study. Data was collected on socio-demographic, health predictors and anthropometric measurements (such as weight, height and waist circumference). The data analysis was performed on JMP®, Version 12. SAS Institute Inc., Cary, NC, 1989–2007.

**Results:**

The prevalence of self-reported chronic pain in Al Kharj population was 19% with a mean age of 26.4 (SD = 8.6) years. The most common locations of pain included; back pain (30%), abdominal pain (26%), headache (13%), and any musculoskeletal pain (56%). Multiple logistic regression revealed that presence of a chronic disease (OR = 3.8; 95% CI = 2.3–6.2), psychological disease (OR = 2.3; 95% CI = 1.2–4.3), high General Health Questionnaire (GHQ)-12 score (OR = 1.06; 95% CI = 1.03–1.1), and pack-years of smoking (OR = 1.05; 95% CI = 1.01–1.08) were significantly related to chronic pain in Al Kharj population.

**Conclusions:**

Our study results found a high burden of chronic pain in this selected Saudi population. The most prevalent pain was low back pain. The presence of chronic and psychological diseases were strongly related to chronic pain. Future prospective studies are needed to establish the temporal relationship of chronic pain with these factors.

## Background

Chronic pain [CP] is defined as pain that persists beyond the normal healing time. With reference to non-malignant pain, 3 months is considered as the point of distinction between acute and chronic pain [1]. Chronic pain is a multidimensional phenomenon which includes; physical, psychological, social and cultural impact on an individual’s health which increase economic burden on health care services [2–4]. Globally, the burden of chronic pain is associated with comorbid medical conditions (such as; diabetes, arthritis, heart disease and cancer) [5].

Chronic pain can be constant or intermittent; that usually persists for a prolong period of time and is not always attributed to a specific cause [6, 7]. It is currently a major public health problem and incurs high cost on an individual as well as on the health system. It is associated with a twofold increase in medical consultations and hospitalization; and five times higher utilization of emergency services [8, 9]. The estimated costs for CP in the United States is more than 600 billion dollars annually [10]. Moreover, according to Krol et al., individuals who suffer from CP often experience deterioration of physical health, social functioning and psychological well-being which results in depression and anxiety [11]. CP affects approximately 20% of the European population with a financial burden of more than € 200 billion per annum [12].

The Global prevalence of CP ranges from 10.1 to 55.2%, with a weighted mean prevalence of 30.3% [13, 14]. Previous studies indicate that the prevalence of chronic pain varies from 12 to 80% [15]. It is widely recognized that the experience of CP is not uniform across countries. The factors that cause this heterogeneity are: Heterogeneous samples, differences in methodologies of the studies, criteria for the time of onset of CP, severity and definition of chronic pain [16]. Moreover, several cultural and psychological variations might also be responsible for such diversity [14, 17]. World Health Organization [WHO] survey suggests a six-fold variation in the prevalence of CP in primary care clinics across countries [18]. For instance, a study conducted in Brazil, reported a prevalence of 42% of CP among middle age individuals and CP had a significant impact on their quality of life. [19]. Moreover, a study from Nepal reported a prevalence of CP of 48–50% while in India it was found to be 24 to 41% [12].

Chronic pain is more common among females and elderly population [12, 17, 19–26]. Vieira and colleagues found lower back pain and headache as the two most frequently reported sites in Brazilian population [24]. Demographic factors associated with CP include; lower socioeconomic status, lower education level, unemployment, and physically demanding jobs [12, 17, 19, 20, 22, 24, 26, 27]. Moreover, geographical and cultural background seems to affect the perception of pain [12]. The psychological factors most commonly reported due to CP include; depression, anxiety, [4, 19, 23, 27] and interpersonal violence [12]. Moreover, obesity, poor self-rated health, marital discord, and smoking may also be related to CP [20, 22]. CP due to musculoskeletal disorders is among the leading causes of years lived with disability [YLD] [28] and one of the most common reasons for seeking medical care [29].

A recent survey conducted in Kuwait found that females have a higher incidence of CP as compared to males and non-Kuwaitis [30]. A review conducted recently indicated an escalation in the prevalence of back pain in the Arab world [31]. In King Khalid Hospital, Hail, Saudi Arabia, the prevalence and patterns of chronic pain due to musculoskeletal system among rheumatology patients was 42%. The most common sites were; low back pain and neck pain with frequency of 52 and 41% respectively [32]. A global pain survey by WHO was published in 1998 but the data from Middle East was not evaluated, therefore, there is dearth of information regarding the prevalence and determinants of chronic pain in the Arab world [18]. Therefore, it is critical to collect information from this population, so that recommendation can be made to the policy makers to allocate resources for relevant healthcare services for improving pain, its related health conditions, and psychosocial well-being. Since previous studies were not population-based and the extent of the problem is not sufficiently known in Saudi Arabia, therefore, this study aims to determine the prevalence of CP and its associated risk factors underlying the health conditions in Al Kharj, Saudi Arabia.

## Methods

### Study design

This is a cross-sectional study design. The participants were recruited from January to June 2016 from Al Kharj, Saudi Arabia.

### Settings

The research was conducted in Al Kharj, which is a city located around 77 km south of Riyadh within the Central region of Saudi Arabia with a population density of 376,000. Al Kharj is an economic hub with significant natural resources, an important geographical location, and population diversity with various racial and ethnic backgrounds.

### Inclusion and exclusion criteria

The selection criteria included; participants of both sexes, aged 18 years or above, who agreed to sign the informed consent. We excluded individuals who were not residents of Saudi Arabia.

### Data collection and sample size/ sampling technique

A multi stage stratified cluster sampling was employed and participants were recruited from different government or private [corporate and educational] institutes. A total of 32 corporate and educational institutes were identified in the region. These institutes were divided into two strata (Government and Private). We selected four public institutes and three private institutes through cluster sampling. Another cluster sampling was done on the selected institutes. Two of these randomly selected institutes included colleges. Sampling units were carefully chosen using simple random sampling from each of the institutes using the list of respondents obtained from each departments of the respective institute. The data was preserved in a password encrypted laptop of the investigator. The data was available to the researcher only due to the principles of confidentiality and secrecy of the respondents. The names of the participants were subsequently allocated, and an individual code/number was assigned, and their names were removed to allow an anonymous selection of the participants. A “computer-generated lists” for random selection of participants was used for generating a list of random numbers.

Our final sample was composed of 1200 participants. After taking informed consent from the participants physical assessment was done. The surveyors did not influence the responses. Participants were free to respond to questions without fear of reprisal. After excluding incomplete data, which was defined as any questionnaire with missing responses to more than 5 questions, a total of 1031 respondents were included in the study.

### Materials/instruments

The questionnaire had several sections. The first section captured demographic profiles such as age, education, employment and marital status. The second section captured pack- years of smoking, chronic medical illness, and chronic pain status. Anatomical areas were illustrated by picture for measuring chronic pain. Responses were recorded using bimodal approach.

We also used general health questionnaire 12. It is one of the most unique and extensively used scale. The GHQ-12 self-reported questionnaire consist of 12 items, each of which are assessed through 4 indexes. Experience of 12 common symptoms is inquired from individuals (such as loss of confidence, and loss of sleep etc.). The two of the most common types of scoring system includes; Likert scoring technique (0–1–2-3) and the bi-modal (0–0–1-1). This is a widely-used instrument designed to screen for psychological distress. The scale inquires if the respondent has experienced any specific symptom or behavior. Each item is rated separately on a four-point scale (less than usual, no more than usual, rather more than usual, or much more than usual) and it gives a total score of 12 or 36 based on the selected scoring method [33]. The GHQ-12 tends to have good specificity, reliability, and reasonably high sensitivity [34, 35]. The validity and reliability of this questionnaire has also been evaluated in Iran [36].

Chronic pain in our study was defined as any self-report of pain lasting 3 months or more [9]. Presence of any comorbid chronic condition was self-reported. The participants were inquired if they had any chronic disease like Diabetes, Hypertension, Heart disease, Kidney disease, cancer, arthritis, liver disease, asthma, COPD or any other chronic disease. In case of an affirmative response, they were given an opportunity to mention/report the specific condition they were suffering from. Smoking was measured by inquiring about the smoking status along with the number of “years and packs” smoked. Physical examination and blood test were conducted followed by the completion of the questionnaire.

### Blood sampling and anthropometry

A blood sample was taken from all participants to calculate Lipid profile mainly HDL (high-density lipoprotein cholesterol) and HbA1c that were measured at the baseline. Evidence suggests that individuals having diabetes (high HbA1c) and high cholesterol are prone to chronic pain. Detailed blood sampling technique has already been defined previously [37].

Weight categories were demarcated following the WHO standard as 30 kg/m^2^ for obese [38]. Waist circumferences (WC); at the level of the hip and umbilicus circumference was measured at the widest girth of the hip using a flexible tape.

### Data analysis

Data analysis was performed using statistical software JMP®, Version 12. SAS Institute Inc., Cary, NC, 1989–2007. Descriptive statistics were reported for categorical variables as frequencies and percentages while continuous variables were reported as Mean (Standard deviation) for normally distributed variables and Median (Interquartile range) for non-normal variables. Simple and multiple logistic regression analysis was used to determine the association of chronic pain with age, years of smoking, weight, waist circumference, HDL, HbA1C, presence of chronic disease and presence of psychological disease. Crude and adjusted Odds ratios and 95% Confidence Intervals were reported and *p*-values of < 0.05 were considered statistically significant.

### Ethical approval

Ethical approval was sought from the “Institutional Review Board” (i.e. Committee of Scientific Research and Publication based in Prince Sattam Bin Abdulaziz University of AlKharj, Saudi Arabia). The participants were informed about the purpose of the research study and were asked to give their written/verbal consent to participate in the study. The participants were also guaranteed of confidentiality of their information and were notified that participation would be voluntary.

## Results

The mean age of the participants was 26.4 years (SD = 8.6) (Table 1). Women were two times more than men (644 vs 387). Majority of the study participants were university graduates (803 /1031: 77.9%). Approximately, 133 participants were either current or ex-smokers. About 84/1031: 8% reported suffering from a chronic disease. Self-reported psychological disease prevalence was only 5% (54/1031).

**Table 1:** Demographic characteristics of the study population

	Frequency	Percentage
	*n* = 1031	(%)
Gender		
Male	387	37.54
Female	644	62.46
Education level		
Primary	22	2.13
Secondary	132	12.80
Intermediate	31	3.01
University	803	77.89
Postgraduate	36	3.49
Occupation (job) status		
Civilian	548	53.15
Not working	33	3.20
Soldier	443	42.97
Marital status		
Married	359	34.82
Single	665	64.50
Smoking Status		
Ex-smoker	31	3.01
Non-smoker	891	86.42
Current smoker	102	9.89
Chronic Disease^a^		
No	940	91.17
Yes	84	8.15
Psychological diseases^a^		
No	970	94.08
Yes	54	5.24
Chronic pain^a^		
No	825	80.02
Yes	198	19.20
Age (years)^c^	26.4	8.6
GHQ score^c,b^	11.88	5.25

^a^Self-reported

^b^General Health Questionnaire 12 score

^c^Mean and standard deviation

The prevalence of chronic pain was 19% (198/1031) in Al-Kharj population (Table 1). Among these, the most commonly reported site of chronic pain was back pain (60/198: 30%) followed by abdominal pain (51/198:26%) and headache (26/198: 13%) (Table 2). Any musculoskeletal pain was reported by half of those with pain (111/198: 56%).

**Table 2:** Location of self- reported chronic pain by anatomical site

Type of pain	Frequency^a^	Percentage
	*n* = 198	(%)
Abdominal pain	51	25.76%
Chest pain	22	11.11%
Facial pain	6	3.03%
Feet pain/ankle pain	14	7.07%
Wrist pain/finger pain	12	4.55%
Knee pain	23	11.62%
Headache	26	13.13%
Neck pain	18	9.09%
Back pain	60	30.30%
Upper back pain	23	11.62%
Low back pain	44	22.22%
Elbow pain/ shoulder pain	12	6.06%
Thigh pain/lower leg pain	17	8.59%
Any Musculoskeletal pain	111	56.06%
Number of chronic pain sites	Mean (SD) 1.6 (0.95)	Median (Range) 1 (1–6)

^a^Each participant could report more than one site of pain

The number of anatomic pain sites ranged from 1 to 6 per subject, while the median number of anatomic sites with chronic pain was 1 (Table 2). The presence of psychological disease was significantly associated with the number of chronic pain sites (*p* = .007).

There was no statistically significant association between the different anatomic sites of chronic pain, sociodemographic, and psychological factors. However, knee pain was found to be more prevalent among participants who had higher mean age (Table 3). Any musculoskeletal pain reported was grouped as one variable, however, the results did not differ from other anatomic sites of pain.

**Table 3:** Characteristics of respondents reporting chronic pain

	Chronic Pain *N* = 198	Any Musculo Skeletal *N* = 111	Chest pain *N* = 22	Knee pain *N* = 23	Headache *N* = 26	Back pain *N* = 60
Age Mean (SD)	28.47 (10.08)	29 (1.09)	27.23 (2.2)	31.83 (14.1)	25.73 (6.9)	28.07 (9.7)
Gender	n (%)*	n (%)*	n (%)*	n (%)*	n (%)*	n (%)*
Female	118 (59.5)	67 (60.4)	12 (54.5)	17 (73.9)	16 (61.5)	38 (63.3)
Male	80 (40.4)	44 (39.6)	10 (44.5)	6 (26.1)	10 (38.5)	22 (36.6)
Marital Status						
Married	75 (37.8)	43 (38.7)	9 (40.9)	8 (34.7)	9 (34.6)	24 (40)
Single	123 (62.1)	68 (61.2)	13 (59.1)	15 (65.2)	17 (65.4)	36 (60)
Job						
Civilian	109 (55)	60 (54.0)	12 (54.5)	11 (47.8)	12 (46.2)	31 (51.6)
Not Working	6 (3)	2 (1.8)	2 (9.09)	0 (0)	1 (3.8)	1 (1.6)
Soldier	83 (41.9)	49 (44.1)	8 (36.3)	12 (52.1)	13 (50)	28 (46.6)
Education level						
Primary	7 (3.5)	6 (5.4)	1 (4.5)	3 (3.0)	0 (0)	3 (5)
Secondary	29 (14.6)	21 (18.9)	3 (13.6)	4 (17.39)	4 (15.4)	8 (13.3)
Intermediate	5 (2.5)	1 (0.9)	0 (0)	0 (0)	1 (3.8)	0 (0)
University	147 (74.2)	77 (69.4)	17 (17.3)	16 (69.5)	19 (73.1)	44 (73.3)
Postgraduate	10 (5)	6 (5.4)	1 (4.5)	0 (0)	2 (7.7)	5 (8.3)
Smoking Status						
Ex-smoker	9 (4.5)	6 (5.4)	1 (4.5)	0 (0)	0 (0)	3 (5)
Non-smoker	167 (84.3)	93 (83.7)	19 (86.3)	20 (86.9)	22 (84.6)	50 (83.3)
Current Smoker	22 (11.1)	12 (10.8)	2 (9.1)	3 (13.04)	4 (15.4)	7 (11.6)

*Percentage in the columns indicates % of respondents having pain out of total individuals with pain experienced at a specific site

Table 4 presents only statistically significant factors identified using multivariate or univariate analysis. Simple logistic regression revealed that predictors of chronic self-reported pain included; age, pack-years of smoking, body weight, waist circumference, HbA1c among others (Table 4). However, presence of chronic disease (adjusted OR, 3.8; 95% CI, 2.3–6.2), presence of psychological disease (adjusted OR, 2.3; 95% CI, 1.2–4.3), pack-years of smoking (adjusted OR, 1.05; 95% CI, 1.01–1.08) and high General Health Questionnaire 12 score (adjusted OR, 1.06;95% CI, 1.03–1.1) were the only predictors which remained significantly associated with chronic self-reported pain on multiple logistic regression (Table 4).

**Table 4:** Predictors of self-reported chronic pain

	Crude			Adjusted		
	Odds Ratios	95% CI	*p*-value	Odds Ratios	95% CI	*p*-value
Age	1.03	1.01–1.05	0.0002	–	–	
Pack years of smoking^a^	1.04	1.007–1.08	0.018	1.05	1.01–1.08	0.017
Weight (kg)	1.011	1.004–1.018	0.0017	–	–	
Waist circumference (cm)	1.009	1.0015–1.016	0.018	–	–	
HDL^d^ (mg/dl)	0.546	0.35–0.84	0.0068	–	–	
HbA1c^e^	1.2	1.03-1.39	0.02	–	–	
Chronic disease^c^						< 0.001
No						
Yes	4.49	2.83–7.13	< 0.0001	3.8	2.3–6.2	
Psychological disease^c^	Reference					0.009
No						
Yes	4.3	2.45–7.53	< 0.0001	2.3	1.2–4.3	
GHQ 12 score^b^	1.07	1.04–1.10	< 0.0001	1.06	1.03–1.1	0.0002

^a^Pack years of smoking = (number of packs smoked/day) X (total number of years of smoking)

^b^General Health Questionnaire score

^c^self-reported

^d^High Density lipoproteins

^e^Hemoglobin A 1C

## Discussion

The prevalence of self-reported CP was 19% in our study and the mean age of the participants was 26.4 years [SD = 8.6]. The most common sites of chronic pain included; back pain followed by abdominal pain and headache and about half of the study population suffering from musculoskeletal pain. Significant predictors of self-reported CP included; pack years of smoking, any chronic or psychological disease, and high General Health Questionnaire 12 scores.

Studies suggest that prevalence of CP varies according to the age group from 14% among 18–25 years old, and 25 to 45% among relatively older age groups [39–42]. Previous literature also demonstrates that the prevalence of chronic pain was higher among older age groups which is contrary to our study results where the mean age of the participants was 26.4 years. The plausible explanation of this could be that Al-Kharj is a new industrial city with a youthful population and age structure in transition. Majority of the population includes people from younger age group. The city has several institutes in the region with a younger working force. A systematic review of 48 studies showed 30% frequency of CP worldwide [13]. However, regional reports of CP prevalence reported from different parts of the world are as follows: 19% in Libya [43], 24% in Iran [44], 56% in Kuwait [45] as a function of participants’ age. Around 40.5% adolescents reported CP in Latvian adults [46].

In agreement with the other studies, we found that the lower back was the most common pain locations as compared to abdominal pain, headache, or any other musculoskeletal pain. About half of our study sample reported musculoskeletal pain; hence demonstrating the highest frequency of pain among Saudi Al-Kharj population. In a recent study conducted by Alnaami et al., the prevalence of LBP among health care workers (HCWs) at different levels of health care in southwestern Saudi Arabia was 73.9% (95% CI: 70.7-77.0) [47]. A study including female Saudi school teachers reported 38% prevalence of severe low back pain followed by knee pain in 26.3, 24% heel pain, 20.6% shoulder pain, and 17.7% upper back pain [48]. This corresponds to our study findings where most of the respondents had CP at similar locations; possibly pertaining to micronutrient deficiencies [49]. Likewise, Wong et al. reported 34.9% occurrence of CP in Hong Kong with higher prevalence in women among old age groups [16]. However, in Latvian adolescents, headache (27.2%) was the most common followed by stomach ache (19.8%) and back pain (13.5%) [46]. However, inconsistency in result can be due to selection of sample, study settings and variation in the area and population of different countries.

Some authors reported CP predictors to be age above 30, psychological illnesses including depression, chronic illnesses [16, 49], which is consistent with our findings. Wong et al. found that chronic illness, increasing age, marital discord, the presence of anxiety and depression and poor quality of life were found to be significantly associated with CP [16]. Likewise, CP was reported more in older, unemployed, and overweight individuals; being highest in the lower extremities of the Kuwaiti individuals [50].

The most important predictor of chronic pain in our study was chronic disease [adj OR = 3.8: 95% CI = 2.3–6.2]. A previous Korean study on data from National Health surveys of 5 years found that the there was a strong association between the presence of chronic diseases, pain severity, and prevalence of chronic pain [51]. The relationship of chronic pain with psychological ill-health is a recognized phenomenon [41, 42, 52, 53]. A study from Dammam explored the relationship between anxiety, depression and chronic pain demonstrating a stronger association of chronic pain among women and younger patients [54]. Pain is one of the somatic symptoms that individuals with psychological issues experience, which is in line with our present study findings. Previous evidence also suggests that individuals having diabetes (high HbA1c) and high cholesterol are prone to chronic pain.

Croft et al. from the UK showed that those with high scores on the GHQ were more likely to present with incident of back pain in the following year as compared to their counterparts [55]. In contrast to a previous study, another study found that with every, one unit increase in GHQ 12 score there was a 6% increased of chronic pain. A study from Japan demonstrated that high scorers on the GHQ showed increased prevalence of body pain even before the appearance of psychological symptoms of ill-health [56]. It was also found that young, and low-risk patients with chest pain seemed to have high GHQ scores [57]. These findings suggest that pain is a complex phenomenon that is influenced by factors like psychological distress.

### Strengths and limitations

The major strength of our study is our robust sampling technique i.e. multistage stratified cluster. All institutes from Al-Kharj were included in the sampling frame. Moreover, we were able to identify predictors of chronic pain and quantify their relationships in the Al-Kharj population. However, this study had a few limitations. Firstly, our study was conducted in Al-Kharj and not in other regions of Saudi Arabia, hence our results cannot be generalized to the whole of Saudi Arabia. However, the study can be generalized to working force of Al-Kharj. Our outcome was self-reported but interestingly it has been found that self-reported pain is more valid than objective measures of pain [58, 59] and has better agreement with medical record data [60]. The findings could be subject to recall bias over a 3-month period for the prevalence of pain and hence only severe nature of pain could be captured which could have led to an under-estimation of pain prevalence. Moreover, it can be argued that the questions on psychological disease and smoking could be subjected to reporting bias.

### Recommendations and implications

Multifaceted public health interventions (social, educational, psychological and behavioral) are suggested to prevent the increase of CP prevalence. A lifestyle free of smoking, weight control and stress-free attitude may help to reduce the development of chronic pain. Individuals should be given opportunities for recreation and physical activity to help reduce the impact of obesity and adopt a physically and psychologically healthy lifestyle. Apart from medicinal treatment, preventive measures and counseling may also impart a significant influence on the management of chronic pain patients. Prospective data is needed in future to establish causality for these associated factors and hence develop targeted strategies to reduce the sufferings of individuals from chronic pain and improve their quality of life.

## Conclusion

Chronic pain has a major impact on an individual’s health. With regards to the pain location, low back pain was the most prevalent site for pain followed by abdominal pain, headache, and any musculoskeletal pain. The presence of chronic disease and psychological disease are strongly related to chronic pain in the Al-Kharj population. Our findings were consistent with other national, regional and international data depicting a high burden of chronic pain. In line with such observations, it can be concluded that chronic pain is preventable. Numerous modifiable factors can conveniently be targeted e.g. smoking, obesity, stress management for public health interventions in order to achieve a peaceful and harmonious life in order to remain aloof from the unhealthy and damaging effects of CP.

## Abbreviations

CP: Chronic Pain
GHQ: General Health Questionnaire
OR: Odds Ratio
WC: Waist circumference
